# Problem Formulation for Off-Target Effects of Externally Applied Double-Stranded RNA-Based Products for Pest Control

**DOI:** 10.3389/fpls.2020.00424

**Published:** 2020-04-16

**Authors:** Alan Raybould, Andrea Burns

**Affiliations:** ^1^Science, Technology and Innovation Studies, Old Surgeons’ Hall, The University of Edinburgh, Edinburgh, United Kingdom; ^2^Global Academy of Agriculture and Food Security, The University of Edinburgh, Midlothian, United Kingdom; ^3^Product Safety, Syngenta Crop Protection, LLC, Durham, NC, United States

**Keywords:** hypothesis testing, problem formulation, acceptable risk, decision-making, targeted risk assessment

## Abstract

Externally applied dsRNA-based biocontrol products may lead to off-target degradation of messenger RNA in target and non-target organisms. For the purposes of regulatory risk assessment of such products, producing a comprehensive catalog of any off-target effects using profiling methods is unnecessary and would be ineffective in supporting decision-making. Instead, problem formulation should derive criteria that indicate acceptable risk and devise a plan to test the hypothesis that the product meets those criteria. The key to effective risk assessment of dsRNA-based biocontrols is determining whether their properties indicate acceptable or unacceptable risk, not whether they arise from on- or off-target effects of dsRNA.

## Introduction

Double-stranded (ds) RNA has roles in virus defense and immune responses in animals (Reich and Bass, 2019) and plants (Niehl et al., 2016). Among other effects, it triggers sequence-specific degradation of messenger RNA (mRNA) via RNA interference (RNAi). GM crops producing dsRNA that triggers RNAi in pests or pathogens are effective in reducing insect damage or disease (Head et al., 2017; Lindbo and Falk, 2017). dsRNA may also be effective against crop pests and pathogens when suitably formulated and applied externally to the crop. Commercial products based on this “non-transformative” technology are in development (Zotti et al., 2017). Biocontrols based on dsRNA are attractive because they are likely to pose low risk to non-target species (Bachman et al., 2016; Joga et al., 2016) and dsRNA has low persistence in the environment (Dubelman et al., 2014; Fischer et al., 2017); however, unintended silencing of transcripts (“off-target effects”) raises concerns (Kulkarni et al., 2006).

Data requirements for regulatory decision-making for biocontrol products based on externally applied dsRNA are not clear (Darsan Singh et al., 2019). Nevertheless, externally applied dsRNA products will almost certainly require assessment of the acceptability of the risks that their use poses to human and animal health and the environment. These risks may arise from exposure to the dsRNA or any formulant that helps the effectiveness of the product (Christiaens et al., 2018). Problem formulation may help in the design and conduct of these assessments.

## Problem Formulation

Problem formulation organizes existing knowledge and identifies relevant new knowledge to support decision-making. Its origins are in ecological risk assessment, but it may be used in any situation where science informs decisions (Sauve-Ciencewicki et al., 2019).

Problem formulation translates policy objectives into operational decision-making criteria and devises tests of the hypothesis that the proposed activity meets those criteria. In regulatory risk assessment, policy objectives are those of the laws that the regulations are intended to implement, and hence are ultimately those of the government of the country making the decision. Policy may be thought of more broadly as the objectives of any decision-maker; thus, our remarks also refer to non-regulatory decision-making, where objectives may be those of non-governmental organizations, such as private companies or public-sector research bodies making product-development decisions. To avoid appearing to advocate particular policies, we do not define specific harmful effects. Instead, we encourage risk assessors to consult policy- and decision-makers to agree definitions of harm before beginning the risk assessment.

Problem formulation also organizes existing data to test hypotheses so that new data are acquired only if the existing data are insufficient for decision-making (Raybould, 2006). Problem formulation is conceptually straightforward, although its implementation is often difficult because the objectives of the decision are unclear or there is uncertainty about how to determine whether taking a course of action is likely to achieve stated objectives.

## Risk Assessment as Hypothesis Testing

Regulatory risk assessments for externally applied dsRNA-based biocontrol products (the dsRNA active substance and any formulants) are likely to draw on experience gained in evaluating uses of biological pesticides (Mensink and Scheepmaker, 2008; Arora et al., 2016), conventional pesticides developed through synthetic chemistry (Finizio and Villa, 2002; Boobis et al., 2008), and GM crops that produce insecticidal dsRNAs (Bachman et al., 2016; Petrick et al., 2016) or proteins (Mendelsohn et al., 2003).

Such regulatory risk assessments work well when they test a hypothesis that directly informs a decision. Risk assessment is part of risk analysis, which can be summarized as follows:

• Use aims of regulatory policy to define what risks are acceptable and unacceptable
• Derive criteria that indicate the proposed product use poses acceptable risk
• Test the hypothesis that the product use meets those criteria
• Use the results of the tests in decision-making about product-use approvals

Problem formulation comprises the setting of acceptability criteria and a plan to test that the criteria are met. Risk characterization evaluates the results of the tests. Problem formulation and risk characterization are the main elements of risk assessment.

A crucial element of problem formulation is the setting of decision-making or acceptability criteria. Toxicity: exposure ratios (TERs) used in making decisions about uses of pesticides are good examples. A TER comprises a measure of the toxicity of a pesticide to a group of organisms and an estimate of the worst-case exposure of that group of organisms when the pesticide is used properly (Damalas and Eleftherohorinos, 2011). If the TER is above a pre-set “trigger” value, risk is acceptable; if the TER is below the trigger, acceptable risk has not be shown. Being above or below the trigger leads directly to different decisions about, for example, whether to require more data to assess risk. In effect, decision-making is based on corroboration or refutation of the hypothesis that the TER is greater than the trigger.

Risk assessment is much less successful when it is data-led. Instead of testing hypotheses about whether certain acceptability criteria are met, data-led risk assessment tests the null hypothesis that the proposed activity will not result in effects that are different from a similar current activity. An example is comparing the effects of exposing organisms to a dsRNA-based biocontrol and to a suitable control substance. Any statistically significant differences are evaluated for their “biological relevance” (EFSA Scientific Committee, 2011). As discussed below, testing a null hypothesis of no difference is a method for accumulating and presenting data, not testing hypotheses that help decision-making, and is an inefficient and ineffective way to assess risk (Raybould and Macdonald, 2018; Raybould et al., 2019).

## Intended and Unintended Effects

In regulatory decision-making about GM crops, risk assessment has been hypothesis-led when considering the potential side effects of the intended modification. Examples include assessing the acceptability of risks posed to biological-control organisms from the cultivation of GM crops with insect-control traits by testing hypotheses about TERs (Romeis et al., 2008), and the acceptability of risks posed to crop production from the cultivation of herbicide-tolerant crops by testing hypotheses about the abundance of herbicide-tolerant weeds (Devos et al., 2018).

Difficulties in GM crop risk assessment have arisen when considering unintended effects of genetic modification (Filipecki and Malepszy, 2006). Instead of using problem formulation to define what unintended properties of a crop would be unacceptable, or at least undesirable, regulatory risk assessments have used a data-led (or “profiling”) approach that tests for statistically significant differences between a GM crop and a suitable near-isogenic non-GM comparator. Many characteristics are compared and the degree of difference in any given character that would indicate unacceptable risk is not predetermined. Comparisons include phenotypic characterization (Horak et al., 2015) and compositional analysis studies (Herman et al., 2017), and some authors have suggested that comparisons are expanded to include transcriptomic, proteomic and metabolomics profiles (Christ et al., 2018). In the remainder of the paper, we use the term profiling mainly to refer to molecular (“omics”) methods, but we intend the term to cover all studies that compare numerous characters without predetermining acceptability criteria; hence, we consider phenotypic and compositional analyses that test null hypotheses of no difference to be profiling.

A similar situation to GM crops applies to products based on external application of dsRNA. A hypothesis-led approach for assessing risks from intended effects could use current frameworks; for example, methods for assessing non-target organism toxicity and exposure to chemical pesticides can be adapted for use with dsRNA-based biocontrol. Adaptation of these methods to dsRNA-based products is considered elsewhere (Sherman et al., 2015; Bachman et al., 2016; Haller et al., 2019) and is not considered further here.

There are suggestions that hypothesis-led approaches to risk assessment should be augmented by molecular profiling of the effects of dsRNA in tissue cultures or standard laboratory test organisms (Heinemann et al., 2011, 2013; Sherman et al., 2015). Proponents of profiling suggest that it will improve human and ecological risk assessment because we do not have a “complete understanding of the biochemistry” of dsRNA-induced RNAi (Heinemann et al., 2011). Others suggest that profiling is unnecessary in many circumstances because negligible risk can be demonstrated based on lack of “functional exposure” to dsRNA because of dietary barriers (Sherman et al., 2015).

The existence of dietary barriers may be a useful hypothesis to test in a risk assessment; however, if the hypothesis that barriers exist is refuted, resorting to profiling of unintended (off-target) effects is still unnecessary. Instead, problem formulation should be used exactly as for the assessment of side-effects of the intended (“on-target”) effects of the dsRNA: devise criteria for accepting that the product use poses acceptable risk and test that those criteria are met. Effects should be judged by their potential to cause harm, not by whether they result from on- or off-target effects.

## Comparing Targeted and Untargeted Assessments

Problem formulation produces a plan to test hypotheses that a product use meets predetermined acceptability criteria. Existing data, and new data if necessary, are sought, or “targeted,” to provide rigorous tests of such hypotheses. In contrast, profiling sets no predetermined acceptability criteria, and aims to describe how a product or its use differs from an existing product or use. Tests of null hypotheses of no difference are used to present the data. As no decision-making criteria are set, all differences are potentially important; hence, data acquisition is untargeted. The differences in philosophy underlying these approaches, their practical implementation and their attitudes to new scientific developments are summarized in Table 1.

**TABLE 1:** A comparison of targeted and untargeted approaches to risk assessment.

**Aspect of risk assessment**	**Untargeted**	**Targeted**
Underlying philosophy	Empiricism	Critical rationalism
Objective	Proof of safety	A tool to support decision-making
Hypothesis tested	No difference from comparator	No unacceptable risk
Number of endpoints	As many as possible	As few as necessary
Decision-making criteria	Sought in the data	Predetermined by policy
Output	Complete understanding	Acceptability of risk
Incorporating scientific advances	Precautionary neophilia	If it ain’t broke, don’t fix it
Resulting decisions	Obscure, arbitrary, disputed	Clear, predictable, accepted

The quality of decisions supported by these approaches differs markedly. Because targeted approaches rely on policy aims and acceptability criteria being set first, they tend to produce clear and predictable, though not necessarily uncontroversial, decisions. In untargeted approaches, acceptability criteria and policy aims emerge only after the data are obtained; hence, decisions may appear arbitrary (Raybould and Macdonald, 2018). Given the undesirable features of decision-making based on untargeted risk assessment, we should examine why it is advocated.

### Philosophy Underlying Risk Assessment

Targeted risk assessment tests hypotheses about concepts such as harm, risk and unacceptability. In regulatory risk assessments, these terms are defined by policy aims and a key part of problem formulation is understanding these aims and translating them into operational acceptability criteria. Untargeted risk assessment avoids operational definitions of harm, risk and acceptability, and instead tests for differences between, say, organisms exposed to dsRNA and those exposed to a control treatment. In using a neutral term like difference, untargeted assessment appears to follow the philosophy of empiricism: the idea that objective knowledge expands by generalizing from observations made without preconceptions (Hahn, 1965).

Targeted risk assessment is more akin to critical rationalism, which postulates that knowledge arises from trial-and-error testing of solutions to problems (Miller, 1994). In critical rationalism, preconceptions (hypotheses) are seen as unavoidable. The targeted approach makes a virtue of operationalizing explicitly value-laden terms, such as harm, risk and unacceptability, to formulate hypotheses directly related to decision-making. Objectivity arises from rigorously testing these hypotheses and disinterestedly evaluating the results. Untargeted approaches imply that risk can and should be characterized objectively without recourse to values, leading directly to “science-based” decisions (Davison, 2010).

A second important philosophical difference is that empiricism sees objective knowledge as a set of truths confirmed by sufficient observations, whereas critical rationalism regards objective knowledge as a collection of tested hypotheses that have not yet been falsified. Targeted risk assessment recognizes that all decisions will contain uncertainty; any conclusion that risk is acceptable is provisional. Untargeted risk assessment, on the other hand, seems to imply that sufficient data will eliminate uncertainty – we can, indeed must, prove that something is safe (has zero probability of causing harm) before we allow its use. These differences lead to risk assessments that vary greatly in their ability to support decision-making.

### Conduct and Use of Risk Assessments

The combination of not defining acceptable risk – because that would be an unwarranted preconception or improper bias – and seeking proof of safety leads untargeted risk assessment to pursue comprehensive descriptions (or “complete understanding”; Heinemann et al., 2013) of products and their potential effects when used. Null hypotheses of no difference between the effects of the proposed product use and a control treatment can be tested. However, such hypotheses do not express an expectation that a new product is no different from existing products; it is a device for presenting data (Stephens et al., 2007). Untargeted risk assessment therefore collects as many data (measures as many endpoints) as time, money and methods allow to make the most complete possible description of the product and its potential effects.

In seeking the best solution to the problem of how to make a good decision, the targeted approach recognizes that risk assessment should test hypotheses that pre-set acceptability criteria are met. It first organizes existing data to test the hypotheses, and only if these data are insufficient for decision-making are new data required. Consequently, targeted risk assessment collects as few data (measures as few endpoints) as necessary for decision-making.

Collecting as many data as possible rather than as few as necessary has bad effects on decision-making. The immediate decision, perhaps whether to approve a proposed use of a dsRNA-based biocontrol, will depend on whether the product has properties that indicate its proposed use poses unacceptable risk. We could think of these properties as needles in a haystack of other information about the product. Problem formulation starts by using policy objectives to define the characteristics of the needles and, based on these characteristics, designs a targeted strategy most likely to find needles should they exist (i.e., rigorous tests of the hypothesis that the product meets acceptability criteria) and keeps the haystack as small as possible. Decisions can be made effectively, because decision-making criteria have already been established (there is little ambiguity about what needles look like), and efficiently, because all data have clear relevance (they help to find needles should they exist).

Untargeted risk assessment deliberately avoids describing needles, and instead builds the biggest possible haystack. In searching through the haystack (testing a null hypothesis of no difference), it will find potential needles (statistically significant differences), but has no means of determining whether they really are needles (indicators of unacceptable risk) or merely peculiar pieces of straw (differences of insufficient consequence to be unacceptable). There is no method for making this distinction until someone defines unacceptability.

Building haystacks and leaving the characteristics of needles undefined increases the likelihood of making a bad decision. Properties of a product that would lead to unacceptable effects if it were used as proposed may be missed in the mass of data produced by the untargeted risk assessment. Hence, in refusing to use problem formulation to define indicators of unacceptable risk, untargeted risk assessment increases the probability of approving a product use that has effects that turn out to be unacceptable. Conversely, beneficial product uses may be refused because differences in a profile are deemed unacceptable based on potentially spurious statistical significance rather than their potential to cause harm.

The consequences of untargeted risk assessment go further than the immediate decision about the product. Time, money and expertise spent reviewing data of unknown relevance about product X cannot be spent evaluating product Y, which may mean that a harmful product receives inadequate scrutiny or that use of a beneficial product is delayed, depending on how decision-makers respond to limited resources. More widely still, the increased costs and uncertainty of decision-making associated with untargeted regulatory risk assessments is a disincentive to produce innovative products that require premarket approvals (Smyth et al., 2014). Hence, untargeted assessments increase risk directly through increasing the likelihood of poor decisions about current products and indirectly by discouraging the development of new, improved products.

### Scientific Advances and Improving Risk Assessment

Regular demands are made for risk assessments to be improved by incorporating new profiling methods (Heinemann et al., 2011; Pott et al., 2018). However, these demands rarely, if ever, give examples of the failure of a specific risk assessment, or what risks are being underestimated. Proponents of the value of new methods of profiling to risk assessment fail to convince because they cannot show how the profiles are a better test of the hypothesis that a product has no properties that indicate unacceptable risk. As profiling is generally associated with a refusal to define unacceptability, this failure is unsurprising. A bigger haystack of data makes no improvement to risk assessment just because the data are acquired using the latest technology.

If the aim of untargeted risk assessment is a complete description (“understanding”) of a product, then new profiling technology must be seen as an improvement because our description would be incomplete without its use. Hence, untargeted risk assessment will be driven by a “precautionary neophilia”; decisions cannot be made without data collected using the latest methods.

Targeted risk assessment has a more skeptical view of new measurement methods. If they provide better tests of the hypothesis that a product has no properties that indicate unacceptable risk, then, all other things being equal, they improve the risk assessment. “Better” may mean that the hypothesis that the product use meets existing acceptability criteria can be tested more rigorously, or that we could test that the product use meets new acceptability criteria should policy aims change. Often, all other things will not be equal; for example, new methods may cost more, take longer or need more expertise to use and interpret than do existing methods. In these circumstances, use of the new method only makes sense if the value of the improvement in the decision outweighs the additional costs.

Setting policy aims must make compromises among different opinions and objectives, and evaluating options to achieve policy aims will be based on imperfect knowledge; hence, no method of decision-making can be perfect. Some people will disagree with the aims of a decision, and the decision may not achieve its aims or may have unwanted consequences, or both. If a decision clearly fails, we should try to correct it and the methods used to reach it should be evaluated and changed if necessary and feasible. However, untargeted risk assessment’s focus on data means that improvements to decision-making would only be sought in technical developments. Use of problem formulation in targeted risk assessment means that improvements to decision-making would also be sought in increased clarity of policy aims and selection of better acceptability criteria. Often it is convenient for politicians to portray risk assessment as completely technical to delay decisions while new data are acquired (Mastroeni et al., 2019). Use of problem formulation and targeted assessment should reduce such sleight of hand.

### Acceptance of Decisions

Increasing the amount of data to support decision-making often increases controversy because supporters of different views are more able to select data that support their argument (Sarewitz, 2004). The heart of this problem is the inability or unwillingness to argue about fundamental values on which opinions are based. Instead, data are used to try to prove that certain opinions are factually incorrect.

The use of untargeted risk assessment poses similar problems. When statistically significant differences are found, they have to be evaluated. Initially, evaluation may comprise a scientific study of the effects of the differences. However, at some point, a decision must be made about whether or not the effects indicate unacceptable risk. In untargeted risk assessment, selection of endpoints based on their ability to indicate unacceptability of risk is deliberately avoided. Consequently, there is no debate about fundamental values that underlie definitions of harm, risk and unacceptability, and so there is no clarity about why certain statistically significant differences are acceptable or unacceptable, and decision-making appears arbitrary. In effect, policy objectives are set by levels of statistical significance, which may be spurious given the numerous endpoints that profiling produces.

Targeted risk assessment should be less prone to such problems. Problem formulation takes the aims of policy and translates them into acceptability criteria. Good policymaking seeks to understand and reconcile opinions based on different values, or at least be clear why it favors one opinion over others. Having such clarity at the beginning means that the results of the risk assessment lead to understandable and predictable decisions. Not everyone will agree with the aims of the decision, but at least the aims will be based on what has been deemed best for society and not on an arbitrary determination of whether a statistically significant difference is “biologically relevant.” Bringing policy disputes to the fore in targeted risk assessment, rather than ignoring them in a futile search for complete understanding, may help to reduce controversies about the use of new technology in agriculture (Carolan, 2008).

## Effective Use of Profiling for Risk Assessment of dsRNA-Based Products

Conceivably, profiling could be useful in risk assessment if an unacceptable profile could be defined by problem formulation. Research may show, for example, that a particular profile in a tissue culture exposed to candidate dsRNAs indicates with high accuracy that the dsRNA would have unacceptable effects in a standard toxicity test using a non-target organism. Whether to replace the toxicity test with a profile would depend on several factors including the reliability and cost of each type of study, and the ethics of continuing to test animals when other options are available (Kroeger, 2006). If a profile were chosen as the decision-making endpoint, its usefulness would come from a prior definition of acceptability, not from an untargeted search for differences. We emphasize that searching for profiles that constitute useful decision-making criteria is a task for basic and applied research and should not be part of product risk assessment.

## Conclusion

Externally applied dsRNA-based biocontrol products may lead to off-target degradation of mRNA in target and non-target organisms. For the purposes of risk assessment, a comprehensive description of any off-target effects using profiling methods is unnecessary. Instead, problem formulation should derive criteria that indicate acceptable risk and devise a plan to test the hypothesis that the product meets these criteria. The key to effective risk assessment is determining whether product’s properties are acceptable or unacceptable, not whether they arise from on- or off-target effects of dsRNA.

## Author Contributions

AR and AB devised and wrote the manuscript.

## Conflict of Interest

AB is an employee of Syngenta. AR was an employee of Syngenta when some of the ideas in this manuscript were developed. Syngenta carries out research and development on biocontrols based on dsRNA.
